# T238. THE ASSOCIATION OF PSYCHOSOCIAL FUNCTIONING WITH BRAIN VOLUME IN THE EARLY STAGES OF (PSYCHOTIC) ILLNESS

**DOI:** 10.1093/schbul/sby016.514

**Published:** 2018-04-01

**Authors:** Renate Reniers, Rachel Upthegrove, Kareen Heinze, Katharine Chisholm, Ashleigh Lin, Beth Charters, Hannah Ball, Graham Hall, Stephen Wood

**Affiliations:** 1 University of Birmingham; 2 Telethon Kids Institute, The University of Western Australia; 3 University of Manchester; 4 Orygen, the National Centre of Excellence in Youth Mental Health

## Abstract

**Background:**

In recent years, psychosocial functioning has received a lot of attention with discussions around its importance in terms of early identification of illness, prediction of outcome, and targeting of treatment. Regardless of diagnostic outcome, both groups of individuals at ultra-high risk for psychosis (UHR) and those with a first episode of psychosis (FEP) show a wide range of functional outcomes. In light of these clinical outcomes, effort has been made to identify neuroanatomical markers for functioning and functional outcome independent of diagnostic status. The present study aimed to increase insight into the association of brain volume with psychosocial functioning in the early stages of (psychotic) illness by investigating the association between grey matter volume and current levels of social and occupational functioning (SOFAS) in healthy individuals, those with emerging mental health problems (EMH), UHR individuals, and those with a FEP.

**Methods:**

Twenty nine healthy controls (12M:17F; mean age 20.97), 27 EMH individuals (6M:21F; mean age 21.24), 31 UHR individuals (14M:17F; mean age 24.40), and 31 FEP individuals (25M:6F; mean age 25.24) were recruited from mental health services, through posters, social media and opportunity sampling, in the wider area of Birmingham, UK. They underwent magnetic resonance imaging at the Birmingham University Imaging Centre and completed the Social and Occupational Functioning Assessment Scale (SOFAS: healthy controls mean 84.41, range 70–95; EMH mean 63.93, range 32–89; UHR mean 54.68, range 35–80; FEP SOFAS 56.65, range 21–95). Images were analysed using the CAT12 toolbox in SPM12. Grey matter volumes were examined controlling for age, gender and total intracranial volume.

**Results:**

Compared to healthy controls, EMH individuals displayed a pattern of grey matter volume reduction in association with reduced functioning scores in medial prefrontal and cingulate areas. The areas spanning volumetric differences between the two groups in their association with SOFAS scores were similar to those identified in previous work investigating the association between brain volume and functional outcome in UHR individuals (Reniers et al., 2016, doi:10.1093/;schbul/sbw086) but were more widespread and disperse. Similar areas of association were observed in UHR and FEP individuals compared to healthy controls but here the pattern was much more specific and more pronounced in the FEP group than the UHR group in the comparison with healthy controls.

**Discussion:**

The present findings provide novel evidence that while those in the early stages of psychotic illness present a unified pattern of association between psychosocial functioning scores and grey matter volume, those with EMH present with a more pronounced but more dispersed pattern, possibly reflecting a more disperse diagnostic outcome. This indicates specificity with psychotic illness in the association between psychosocial functioning and brain volume and suggests importance concerning our ability to predict outcome and target interventions. In addition, it provides support for the recent focus on functioning in addition to distinct diagnostic categories.

